# Editorial: Oral Nanotherapeutics for Colon Diseases

**DOI:** 10.3389/fbioe.2021.816304

**Published:** 2021-12-09

**Authors:** Lian Duan, Fan Zheng, Didier Merlin, Hangxiang Wang, Zeyu Xiao, Bo Xiao

**Affiliations:** ^1^ State Key Laboratory of Silkworm Genome Biology, College of Sericulture, Textile and Biomass Sciences, Southwest University, Beibei, China; ^2^ Digestive Disease Research Group, Institute for Biomedical Sciences, Georgia State University, Atlanta, GA, United States; ^3^ Atlanta Veterans Affairs Medical Center, Decatur, GA, United States; ^4^ The First Affiliated Hospital, Key Laboratory of Combined Multi-Organ Transplantation, Ministry of Public Health, School of Medicine, Zhejiang University, Hangzhou, China; ^5^ Department of Pharmacology and Chemical Biology, and Institute of Molecular Medicine, Shanghai Key Laboratory for Nucleic Acid Chemistry and Nanomedicine, Renji Hospital, Shanghai Jiao Tong University School of Medicine, Shanghai, China

**Keywords:** oral administration, inflammatory bowel disease, colon cancer, nanopartcicles, treatment

Colon diseases are emerging as a global health threat with rapidly increasing incidence. The most common colon disease is chronic and recurrent inflammatory bowel disease (IBD), with clinical manifestations that include weight loss, mucopurulent bloody stools and diarrhea. Recurrent IBD is currently non-curable and has been proven to increase the risk of colon cancer, which is one of the most serious malignancies with approximately 1.8 million new cases per year and 0.9 million deaths globally. The precise etiology of IBD is still controversial, but some factors such as imbalance of intestinal microbiota, intestinal epithelial barrier dysfunction and immuno-regulatory disorders, are considered to contribute to its initiation and development.

Steroids, anti-inflammatory agents and immunosuppressive drugs have been used to treat IBD, but their therapeutic efficacy is far from satisfactory, and the adverse effects of these medications seriously restrict their long-term use, resulting in poor recovery of normal bowel function. In practice, half of the patients undergo operations to remove pathological tissues within a decade of diagnosis. Chemotherapy is the most common treatment strategy for colon cancer, but success is limited by rapid drug clearance, development of drug resistance and adverse side effects.

The oral route has been widely used in the treatment of colon diseases, which is the most acceptable approach because of its convenience for self-administration and direct delivery of drugs to the pathogenic sites in the colon. However, the development of nanotechnology has provided new types of oral drug delivery platforms. Oral nanotherapeutics can be constructed to contain a specific drug payload with the ability to resist the harsh environment of the gastrointestinal tract, penetrate through the mucosal barrier and target the diseased intestinal areas to provide highly effective treatment for IBD and colon cancer.

In the Research Topic “*Oral nanotherapeutics for colon diseases*”, inspiring oral nanoplatforms were designed for treatment. Current trends and predicaments for the diagnosis and treatment of colon diseases were also been discussed.

Crohn’s disease and ulcerative colitis are the main clinical forms of IBD. Some colon diseases such as intestinal tuberculosis (ITB) are difficult to distinguish from IBD because of similar symptoms, but this must be done because their therapeutic strategies are completely different. He et al. reported that the gut microbiota in patients with ITB was remarkably different from that of Crohn’s disease patients. The microbiota in the gut of ITB patient was characterized by mucosa-associated organisms such as *Proteobacteria*, while an increased abundance of *Bacteroides*, *Faecalibacterium*, *Collinsella* and *Klebsiella* were the features of Crohn’s disease. These results suggest that gut microbiota analysis might be a convenient tool for differentiating ITB from Crohn’s disease.

The therapeutic outcomes of oral nanotherapeutics for colon diseases are strictly dependent on their delivery efficiencies of drugs to the target cells. Lin et al. fabricated natural lentinan-based nanoparticles and used them to deliver drugs to ulcerative colitis lesions. These nanoparticles not only encapsulated the anti-inflammatory drug (budesonide) with high efficiency, but also facilitated the specific uptake of budesonide by macrophages via the Dectin-1 receptor, which increased the drug concentration in the inflamed colon. Further studies demonstrated that the anti-inflammatory activity of these budesonide-loaded nanoparticles was attributable to inhibition of the TLR4/MyD88/NF-кB signaling pathway. Xie et al. produced pluronic F127-modified, electrospun fibrous meshes to encapsulate the hydrophobic drugs, camptothecin and curcumin. The drug release results revealed that these two types of drugs could be released simultaneously and sustainably from the meshes. The released drugs exhibited strong synergistic anti-colon cancer capacity, reinforcing the potential of pluronic F127-modified electrospun meshes for localized combination chemotherapy of colon cancer.

In recent years, a number of oral nanoplatforms have been utilized in the treatment of colon cancer. Sabra et al. summarized the design philosophy of different types of oral formulations, including ROS-responsive nanotherapeutics, prodrugs, time/pH-dependent nanotherapeutics and microflora-activated nanotherapeutics. They concluded that oral formulations had the advantages of convenience and high drug delivery efficiency and therefore might be a better choice than traditional formulations via intravenous injection or rectal administration in colon cancer therapy. However, some issues still remain to be solved before clinical applications can be approved, including the expense of research and development and the difficulty of large-scale manufacturing. Zheng et al. discussed biologics and the existing formulations for clinical IBD therapy. They stated that the oral route was a promising strategy for the treatment of IBD, for reducing adverse drug reactions and increasing local drug concentrations. However, the complexity of these oral formulations, as well as the expense and time involved in testing them on animal models, restricted their translational application. Currently, only four oral biologic formulations have been tested in clinical trials. Unfortunately, none of them has been approved by the Department of Pharmaceutical Administration. The application of oral therapeutics in colon cancer treatment faces a similar predicament. Ying et al. reviewed the features of different oral nanotherapeutics for the treatment of colon cancer, and pointed out that these nanotherapeutics were beneficial because of their stability, controlled release and enhanced localized drug concentration in the tumor. Nevertheless, the clinical application of oral nanotherapeutics for colon cancer therapy was still limited by problems with mass production and overall cost-effectiveness apart from biosafety and pharmacokinetics. Strategies to reduce the complexity of nanoplatforms and optimize dosage forms have to be tested to accelerate the clinical translation of these formulations.

These research studies and reviews provide an overview of the latest and most exciting advances in the application of oral nanotherapeutics to the treatment of colon diseases. In addition, the discussion of this topic may help readers better understand the latest developments and existing problems in this fast-moving research field.

